# Review on Wearables to Monitor Foot Temperature in Diabetic Patients

**DOI:** 10.3390/s19040776

**Published:** 2019-02-14

**Authors:** Jesús Martín-Vaquero, Ascensión Hernández Encinas, Araceli Queiruga-Dios, Juan José Bullón, Alfonso Martínez-Nova, Jose Torreblanca González, Cristina Bullón-Carbajo

**Affiliations:** 1 Department of Applied Mathematics, University of Salamanca, E37008 Salamanca, Spain; ascen@usal.es (A.H.E.); queirugadios@usal.es (A.Q.-D.); 2 Department of Nursing, University of Extremadura, E06006 Badajoz, Spain; podoalf@unex.es (A.M.-N.); cristinabulloncarbajo@gmail.com (C.B.-C.); 3 Department of Chemical and Textile Engineering, University of Salamanca, E37008 Salamanca, Spain; perbu@usal.es; 4 Department of Applied Physics, University of Salamanca, E37008 Salamanca, Spain; torre@usal.es; 5 ETSII Béjar, E37700 Béjar, Spain; 6 Centro Universitario de Plasencia, E10600 Plasencia, Spain

**Keywords:** diabetic patients, foot temperature, medical devices, health monitoring

## Abstract

One of the diseases that could affect diabetic patients is the diabetic foot problem. Unnoticed minor injuries and subsequent infection can lead to ischemic ulceration, and may end in a foot amputation. Preliminary studies have shown that there is a positive relationship between increased skin temperature and the pre–ulceration phase. Hence, we have carried out a review on wearables, medical devices, and sensors used specifically for collecting vital data. In particular, we are interested in the measure of the foot–temperature. Since there is a large amount of this type of medical wearables, we will focus on those used to measure temperature and developed in Spain.

## 1. Introduction

Diabetes mellitus describes a group of metabolic diseases in which the patient has high blood glucose (blood sugar), either because insulin production is inadequate, or because the body’s cells do not respond properly to insulin, or both. If left untreated, diabetes can cause many complications. Acute complications can include diabetic ketoacidosis, hyperosmolar hyperglycemic state, or death. Serious long-term complications include cardiovascular disease, stroke, chronic kidney disease, foot ulcers, and damage to the eyes. Diabetes is a major contributor to cardiovascular diseases and is the eleventh common cause of disability worldwide.

Several different studies mention a significant increases in diabetes rates: 422 million people in 2014, according to the World Health Organization (WHO), or around 425 million people in 2017 [1] had diabetes worldwide. This represents 8.3–8.5% of the adult population [2] (in 1980 was around 4.7%), with equal rates in both women and men [3]. The amount of diabetic patients in Europe was 58 million. This will rise to 67 million by 2045. In 2017, there were 3584.5 people suffering from diabetes in Spain (this represents the 3rd country in Europe) [1]. The global economic cost of diabetes in 2014 was estimated to be US$ 612 billion [4], and US$ 727 billion in 2017 [1].

Several studies have been conduced related to therapeutic footwear with the objective of prevention of reulceration in patients with diabetes and foot risk factors, but no significant protective benefits have been found so far [5]. The chronic illness care leads us to work on patients’ self–management skills and also in tracking devices to improve the quality of diabetic patients life. However, the patients should be responsible of their own health care [6].

Research estimates that the lifetime incidence of foot ulcers within the diabetic community is around 15% and may become as high as 25% [7]. The prevalence of ulcers varies according to sex, age and population from 2.4% to 5.6%. It is a frequent cause of hospitalization and could lead to major complications [8]. Actually, a diabetic foot can be defined as infection, ulceration and/or destruction of deep tissues associated with neurologic abnormalities and various degrees of peripheral vascular disease in the lower limbs [9]. In addition to diabetic peripheral neuropathy (DPN) and peripheral vascular disease, a diabetic foot ulcer may be caused by minor foot trauma, foot deformity and decreased tissue perfusion [10]. Around half of the patients with a diabetic foot ulcer have co-existing peripheral artery disease. Where wounds take a long time to heal, infection may set in and lower limb amputation may be necessary. Foot infection is the most common cause of non-traumatic amputation in people with diabetes. Actually, it is estimated that about 85% of diabetics suffering from amputations have previously had an ulcer [11].

Although DPN is common and a frequent cause of morbidity and disability, early evaluation and treatment of diabetic neuropathy is often neglected and forgotten by patients [12].

Some results found the correlation between the values of vibration perception threshold (i.e., diabetic neuropathy) and the mean foot temperature (MFT) [13]. However, they did not find correlation between glycated hemoglobin (HbA1C) and MFT. Moreover, diabetic patients with neuropathy had higher mean values compared to non-neuropathic subjects. These authors stressed the importance of temperature monitoring to avoid or reduce foot ulcers in diabetic patients.

Prevention of diabetic foot may include optimizing metabolic control (regulating glucose levels); identification and screening of people at high risk for diabetic foot ulceration; and patient education in order to promote foot self-examination and foot care knowledge. Patients would be taught routinely to inspect their feet for hyperkeratosis, fungal infection, skin lesions and foot deformities. Control of footwear is also important as repeated trauma derived from tight shoes can be a triggering factor. However, there is only limited evidence that patient’s education would have a long-term impact as a preventive measure.

Actually, different clinical methods used to assess small fiber function can be classified into electrical contact thermometry, cutaneous temperature discrimination thresholds, infrared thermography, and Liquid Crystal Thermography (LCT). Infrared (IR) thermography has been used as a real-time temperature measurement technique, used to produce a colored visualization of thermal energy emitted by the measured site at a temperature above absolute zero. Jones and Plassmann [14] provided an excellent review on IR technology along with related image-processing considerations.

However, as far as authors know, there is no efficient medical device to alert patients in order to avoid ulceration in diabetic patients. This study is organized as follows: Section 2 includes the art state related to medical devices to monitor patient’s vital constants; the specific case of monitoring body temperature is in Section 3, and a review on the Spanish scientific literature about smart devices for collecting temperature data is detailed in Section 4.

## 2. Devices to Monitor Patient’s Health

The applications on wearable devices to facilitate and improve the quality of life of patients with different pathologies have been a reality for several years, and they have rapidly evolved. However, their clinical efficiency is still debateable.

For instance, Tamura et al. [15] tried to improve the quality of life of elderly and disabled people monitoring at home without restriction during sleep or bathing. Data were automatically collected without any problem from monitoring device.

In 2010, López et al. [16] presented a new pilot scheme developed for real users employing a combination of e-textiles and a wireless sensor devices that control some parameters like body temperature and electrocardiogram (ECG), among others. This combination would provide efficient and noninvasive healthcare-monitoring with the use of a wearable system, in such a way that patients were monitored in real time without having to go to the hospital.

In [17], a system to enable home monitoring for patients with Parkinson’s disease was proposed. This system includes a wearable sensor that collects data, and a two-way communication web application between the patient and the doctor.

In 2013, a wide range of wearable systems was studied, including their applications and effectiveness both at home and in a hospital environment [18], reaching the conclusion that, although they are good and beneficial, they are sometimes limited and do not achieve the precision and safety required by medical staff.

In recent years, mobile health and sport and training monitoring systems have increased the awareness and popularity of wearable technologies considerably [18]. Several multisensory systems to collect physical activity have been developed. The use of the mobile phone to collect sensors data using a low energy Bluetooth communication module is available and easy to use for most of the people [19].

In the Spanish eHealth Report [20], it was stated that “According to the World Health Organization (WHO), the official definition of eHealth is as follows: eHealth is the secure use of information and communications technologies in support of health and health-related fields, including healthcare services and processes, prevention, health surveillance, treatment, health literature and health education, knowledge and research. eHealth can help cut costs and also includes a high sales potential. The field of eHealth is wide, and it is not simply possible to cover all areas that represent the eHealth market in its entirety”. In fact, they report that, in 2017, 19% of the purpose of eHealth apps is to measure body functions.

These eHealth devices are of special interest for chronic diseases, since they would allow patients to have fewer visits to the doctor/hospital with the consequent improvement in the autonomy of life, while health expenses are reduced. The use of wearables for health care will allow for reducing medical costs (reducing hospital stay), will provide access to care and to specialized physicians anywhere and anytime, will prevent diseases, etc. [21].

There are actually several chronic diseases that make patients continuously monitor their health. The education and control could considerably reduce the risk of medical complications. The out-of-hospital quality of life becomes a challenge for physicians and researchers. Many medical wearables to monitor different healthy variables have been implemented to involve patients in their own health care. Wearables specifically designed for human gait analysis [22], diseases such as heart-attacks, sleep apnea, Parkinson disease [23], or elderly people activity (all the physiological signals and physical activities of patients) could be monitored with the help of wearable devices [24,25,26]. Some wearables are available to assess gait. One of them analyzes foot plantar pressure, inertial measurement, laser distance sensing and electromyography [27]. Others could help to retrain gait and help for the functional rehabilitation of patients with Parkinson disease [23,28,29]. A large amount of similar medical wearables, and papers developing them can be found at webpages such as https://www.mc10inc.com/our-products.

Smart or intelligent textiles are playing a significant role in the area of health monitoring. As an example, we could cite the case of fabrics with metallic threads that give information about different body variables, or clothes that measure the heart rate, temperature, and breathing [30]. Reddy et al. developed and analyzed [31] an insole to take measures of temperature in diabetic feet to study the etiology of diabetic foot ulcerations. This system has four sensors at four foot locations in the insole: the hallux, between the first and second metatarsal head, the lateral side of the foot, and the heel.

The variables that are measured by the medical wearables include mainly the following: ECG, respiration rate, oxygenation of the blood (SpO${}_{2}$), photoplethysmogram (PPG), galvanic skin response (GSR), body temperature, and blood pressure. A detailed description of some wearables that have been developed is included in Table 1. The end users of such devices are soldiers, firefighters, police, senior citizens, infants, athletes, or patients in general [32].

The AgaMatrix iBGStar blood glucose monitoring system that includes a glucometer connected to a mobile App (http://agamatrix.com/), or the Proteus digital health system, allow a continuous monitoring related to pills consumption. Diabetic patients also have the possibility of pricking their fingers with a lancet (at home), taking a laboratory card for the blood, mailing it for analysis, and viewing the results (including hemoglobin, hematocrit, glucose, potassium, calcium, pH, urea nitrogen, etc.) on the web [33].

Some other studies have been carried out to examine the effect of therapeutic footwear on ulcer outcomes in diabetic patients, and therapeutic footwear were developed to prevent foot ulcers in at-risk patients [5] and references therein. Otero et al. [38] presented, both at the level of research as industrial and market, an overview in relation to the incorporation of biomedical devices to telecare systems. Patel et al. [39] presented a novel wearable computing platform for unobtrusive collection of labeled datasets and a new paradigm for continuous development, deployment and evaluation of machine learning models to ensure robust model performance. Furthermore, Lonini et al. [40] described machine learning algorithms that use data streams captured from soft wearable sensors and have the potential to automatically detect Parkinson’s disease symptoms and inform clinicians about the progression of the disease.

## 3. Smart Technology to Continuously Monitor the Body Temperature

To analyze the main factors of the increase or decrease of the feet temperature in several points, it might be interesting to design and develop a wearable prototype to obtain foot temperatures continuously, and to alert the patient if there is a risk of ulceration. Obviously, before this prototype is developed, we should analyze the performance of different sensors and see (i) how they act in under a variety of conditions, and it will also be necessary to decide (ii) how many sensors are needed and where we are placing them. This is described in this section and all along the following one, where Spanish research about these topics is described.

In 2017, the estimated quantity of wearable sensors for healthy–care devices was 80 million [33]. Temperature is one of the most measured physical quantities. Temperature sensors transform a physical quantity into an electrical voltage. However, characteristic curves are not always linear, hence electronics must make corrections to obtain the highest possible precision. The use of a sensor for temperature measurement has been discarded in some cases because temperature data can vary depending on environment conditions [34]. In Table 2, we have detailed some of the medical wearables specifically designed to get temperature data.

Lavery et al. [43] defined a method for monitoring skin temperature at predetermined locations on the body, such as the bottom of the feet, to predict foot problems such as ulcerations or any other affliction that cause tissue inflammation and injury. The device implemented by Lavery et al. detects significant differences between the skin temperatures on the bottom of the left foot and the bottom of the right foot, and also a difference between adjacent points. In case of it occurring, an alarm signal is provided to the user. The collected temperature data can also be displayed to the user, stored for future use, or sent to any other device.

Tamura et al. used 16 thermistors PBN-41E to develop a data logger with a memory card to measure temperature while the person is sleeping [41]. The measurement range was 0 to $40{\;}^{\circ }$C with an accuracy of $\pm 0.2^{\circ }$. A multipoint temperature logger using LM35 as sensor and Arduino UNO to collect environmental data was developed [42], with a temperature Range of 0 to $100{\;}^{\circ }$C.

There is a wide number of manuscripts in the scientific literature describing different procedures to monitor the temperature continuously in several points of the human body with non-invasive techniques [44,45,46]. They all try to obtain accurately the core temperature [47], or cerebral temperature [46] and references therein, or ear temperature [48], or rectal temperature. In some cases, they compared several different approaches [49].

Although rectal temperature assessment is the best to be close to the central core temperature, the thermoregulatory function of the skin contributes to its control. Hence, the relationship of body core and skin temperatures in the thresholds of thermoregulatory response is linear [50]. Usual diabetic foot screening includes measurement of infrared skin temperature in the temple (temporal artery) area, but with small, cheap and easy to use devices [51]. In addition, if this device is used in the foot surface, it offers only one measurement data in a certain point, with no accurate results. The protocol that we propose, more accurate, complete and reliable, shows multiple temperature points, with an accuracy of $\pm 0.045{\;}^{\circ }$C.

All these procedures described above utilize many different types of temperature measurement devices such as: (i) Electrical devices as thermocouples, thermoresistance, thermistors, diodes or programmable electronic devices; (ii) Mechanical devices: dilation systems, glass thermometers with liquids, bimetallic thermometers; (iii) Devices with thermal radiation using infrared radiation or thermography; and (iv) Other devices: with color indicators such as pencils or paints, pneumatic probes, ultrasonic sensors, pyrometric indicators or acoustic thermometers [52,53,54]. Without any doubt, electrical sensors are the ones most commonly used for temperature measurement. However, each of these types of sensors have special qualities that make them more convenient for a specific process or goal (see [55] for the description of different types of sensors and processing elements used in various systems of temperature measurement and control).

Thermocouples are the most commonly used electrical temperature sensors in the industry. The classification of thermocouples according to the material and the voltage that they generate is detailed in Table 3. A thermocouple is the union of two wires of different material at a point. When temperature is applied in this point, a very small voltage is generated (of the order of micro or millivolts). If we work with small tension, then it is not easy to get temperature data.

Thermoresistance works by varying its resistance as a function of temperature. The most common devices are built with a platinum resistor called PT100, PT1000, etc. The temperature resistance ratio corresponding to the platinum wire is so reproducible that the platinum resistance thermometer is used as the international temperature standard. Other materials such as nickel, nickel–iron, copper and tungsten are also used. The measurement of this type of sensors must be done with special conditions to achieve greater precision; these measures are called two-wire, three-wire and four-wire, the latter being the most accurate in terms of measurement, but the most complex to perform. A very common resistance sensor is the PT100. This sensor is made of platinum and has a resistance of 100 Ω at $0{\;}^{\circ }$C, varying 0.39 Ω per degree centigrade increased or decreased.

Thermistors are much more sensitive, composed of a synthesized mixture of metal oxides. The thermistor is essentially a semiconductor that behaves like a “thermal resistor”. They can be found on the market with the denomination NTC (negative temperature coefficient, that is, the resistance decreases with temperature) and PTC (positive temperature coefficient, that is, the resistance increases with temperature). NTCs are made of a mixture of Mn, Ni, Co, Cu, and Fe oxides and they are molded into ceramic bodies of different sizes. They typically have a resistance between 50 Ω and 1 mΩ at $25{\;}^{\circ }$C, and a sensibility of 4% at $25{\;}^{\circ }$C. PTCs are resistances that are mainly composed of barium and strontium with titanium. The SSTENTC1K resistance, for example, is very well documented by the manufacturer. The resistance of 1 kΩ has less temperature deviation; specifically, it is 1%, while the others suffer more variation. In addition, this deviation in the measurement of temperature occurred in the vicinity of temperatures close to that of the human body. At $25{\;}^{\circ }$C, we have a resistance of 1 kΩ. Thermistors are much easier to use than the previous ones; it is enough with a simple voltage divider. The equation that dominates the resistance change of a thermistor with respect to temperature is given by the equation:

$$R\left(T\right)=R\left(T_{0}\right)e^{k\left(\frac{1}{T}-\frac{1}{T_{0}}\right)},$$

where R(T) is the resistance (in ohms) observed at the temperature *T*, which depends on a first parameter given by the resistance at a known temperature, $R(T_{0})$, and a second adjustment parameter *k* [53]. However, thermistors are sensitive devices because, if the additional heat can not be dissipated, the heating caused by the current excitation may increase the temperature of the sensing element.

Programmable electronic devices are the latest generation devices to measure temperature. They are already integrated circuits where the temperature variation is made electronically, like the diodes, by voltage and current variation in the PN junction of the semiconductors. The great difference of these is that they are already encapsulated in very small elements and that they communicate directly with a microprocessor to know the temperature around them. There is a great variety of models, typical examples are the MAX30205, the Si7006, the AD590, etc.

Of all those described above, each one has its advantages and disadvantages. For measuring foot temperature, a sensor that can be read easily is necessary, and it must be precise and as small as possible. However, there is not a large amount of literature about techniques to continuously monitor the foot temperature. This is probably caused for several reasons: this sensor must be very small and comfortable, but still precise enough during several months and after several washes. Recently, in [56], how a thermistor chip can be embedded into the fibres of a yarn is explained, and this can be used to produce a textile or a garment. At the same time of the development of this preliminary work, a proposal of a wearable sensor-based system was introduced for foot temperature monitoring in [57]. However, it is necessary to continue with these type of projects if the final goal is to build an efficient medical device which alert diabetic patients when ulcers appear in their feet.

## 4. A Review of eHealth Monitoring Systems in Spain

The use of sensors began more than 200 years ago; in the first industrial revolution, physical sensors were used for pressure control in steam engines and later chemicals and biochemicals applied them to different processes and applications. They were also used to convert a mechanical signal into an electrical signal, which allowed the information captured by the analog electrical sensors to be digitized, thus allowing their subsequent digital processing.

The term “smart” or “intelligent sensor” first appeared in technical literature in the early 1980s with the aim of integrating a digital processor and its software into a physical sensor. This system would allow in situ processing of sensor information and decision making for process control [58]. In their evolution, “smart sensors”, connected in a network, are capable of exchanging data and producing feedback if necessary. Today, they are not only capable of reproducing temperature and humidity conditions, but they are also able to detect applications such as electrical impedance, voltage, magnetic fields, light, chemical or gas concentration, radioactivity and many other measurable conditions. In addition, smart sensors can determine their own physical location and motion (acceleration, speed and vibration), which are used for object detection and to track applications.

Due to advances in miniaturization, most objects or products can be equipped with a smart sensor. In manufacturing processes, intelligent sensors help to monitor, control and improve automated operations. This can be done by detecting the exact position of products and tools, by measuring their dimensions and contours during production and by delivering products to customers. Moreover, these devices enable predictive maintenance of both facilities and products; and they add value to physical objects such as vehicles, aircraft or medical devices [59,60].

Low energy consumption is a key factor in this context of sensor devices, which will require the development of new materials and production techniques. It is also expected that, in the future, sensors will be able to measure new physical, chemical or biological properties [61,62]. For example, neuronal sensors that collect different types of brain or physiological signals, which will trigger future applications in the healthcare sector. In the food industry, the capacitive of detection of microorganisms can be used to check the quality and safety of food.

In the European Patent Office (EPO), we can obtain patent databases, which contain the latest technical information, much of which cannot be found in any other source (https://www.epo.org/index.html).

**Figure 1:**
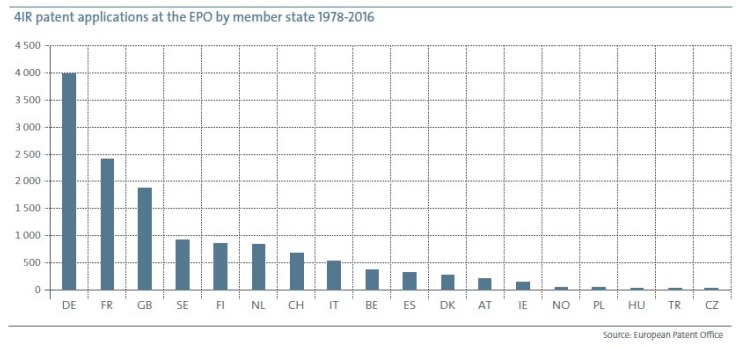
Number of 4IR patent applications at the EPO by member states during the period 1978–2016.

European inventors were responsible for more than 14,000, almost 30%, of all fourth industrial revolution (4IR) patent applications at the EPO up to 2016. Germany contributed with approximately 4000 inventions, the largest in Europe, followed by the other two big European countries: France and the United Kingdom, with more than 2400 and 2000 patent applications, respectively (see Figure 1 with the detailed information). Behind the top three countries, there is a group of others developing significant innovative activities in technologies related to Industry 4.0 (the massive deployment of the Internet of objects has prompted a new industrial revolution): the two Scandinavian countries, Sweden and Finland, and the Netherlands, with approximately 900 patent applications each one. Switzerland is not far behind. Italy and Spain, two other big European countries, are 8th and 10th, respectively.

**Figure 2:**
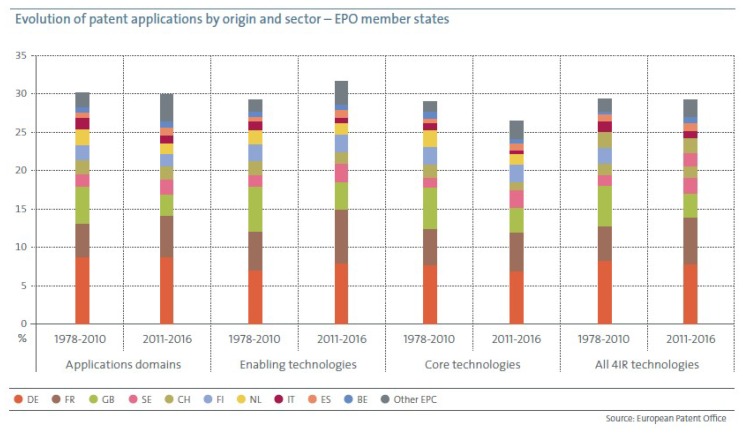
Contributions of each of the EPO member states to European participation in the application domains, and enabling and core technologies related to Industry 4.0 between 1978–2010 and 2011–2016.

Figure 2 shows the contributions of each of the EPO member states to European participation in the application domains, enabling technologies, and core technologies related to Industry 4.0 between 1978–2010 and 2011–2016.

With approximately 60% of all European inventions, Germany, France and the United Kingdom were the three largest inventors in the three sectors in both periods. Spain is another country that shows a positive development. It expanded its participation in application domains and enabling technologies and more than doubled its core technologies.

These statistics analyze the fast growth in the number of patent applications in 4.0 technologies in recent years. In 2016, more than 3% of all applications filed in the EPO combined the characteristics of computing, connectivity, smart objects and data exchange that define inventions 4.0. This fact indicates the considerable potential of connected objects that operate autonomously.

The Spanish Patents and Trademarks Office (SPTO) is the public body responsible for the registration and granting of the different types of industrial property. The SPTO has a unique volume of technological and commercial information in Spain for its content, since it includes all patent documents, utility models, industrial models and drawings, industrial designs, trademarks and other distinctive signs.

The data included below were extracted from the SPTO database. Both documents of patents and utility models have been obtained using specific queries to the database that belong to these institutions in EPO http://www.oepm.es/en/index.html.

During 2017, a total of 2286 patents were filed at the SPTO (filed by residents of the autonomous communities and non-residents). The number of national patents on 31st December 2017 was 37,866 and the number of concessions made in 2017 reached 1944 granted files.

**Figure 3:**
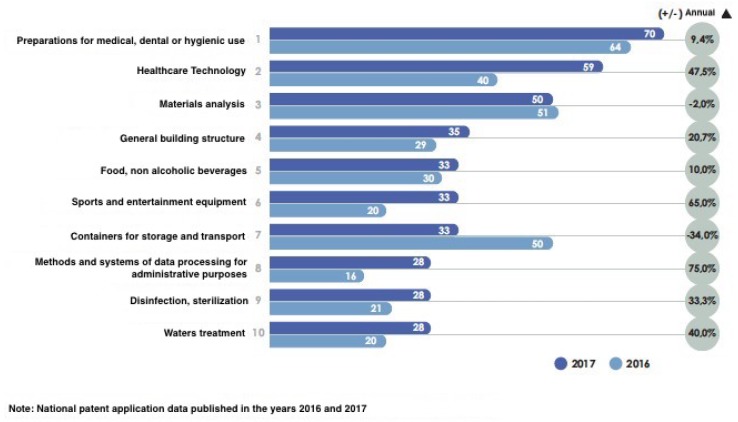
Number of published Spanish patents classified according to their area of application.

In Spain, during 2017, the largest number of published national patent applications was concentrated on technologies collected in the 10 main groups of the International Patent Classification (IPC): Preparations for Medical, Dental or Toilet Use, Medical Technology and Materials Analysis, etc. (see Figure 3). The technologies with the greatest increase are those related to data processing methods and systems for administrative purposes (75%), sports equipment and entertainment (65%), and medical technology (47.5%). On the other hand, the greater decrease, with 34%, corresponds to the sector of containers for warehouse and transport.

In Spain for the same year, 2017, the largest number of requests for national utility models published were concentrated in technologies collected in the 10 main groups of the IPC.

Looking at the evolution between 2016 and 2017, we can see that the booming technologies for protective clothing, outerwear (25.9%) and sports equipment and entertainment (13.8%) have shown an increase; however, there has been a significant decrease in the technologies corresponding to office furniture (30.6%) and poultry, fish and fishing (19.6%).

Thus, it is clear that the use of wearable technology and eHealth products is growing in Spain. In fact, there is no good translation for “wearable”, so this word is widely used as part of the Spanish vocabulary. The use of medical e-textiles was included by Nuubo (https://www.nuubo.com) to develop a biomedical shirt that collects data from electrocardiographic signal through the textile electrodes technology (BlendFix^®^) integrated into the garment [63].

An example of a Spanish patent and utility model in the field of health care is the children’s temperature measuring pacifier [64]. The author developed a pacifier that comprises a body with its ring and extreme ring as well as the teat. This invention is characterized by the fact that the body is fitted with a temperature sensor arranged inside the teat connected to an electric battery located in a housing of the soother’s body, on whose body appears a digital reading screen from which the corresponding temperature is measured. A similar device was patented in [65]. In this case, the authors proposed a pacifier that detects the temperature of a baby who, in contact with it, whether in the oral cavity, hand or body, and in the event of an increase in body temperature, the device changes color due to being made of a specific sensitive material.

Vidal Solà et al. [66] patented children’s pajamas with an integrated temperature sensor, characterized by the fact of understanding, housed internally in the armpit area, a temperature sensor connected to a control unit which, by means of a mini radio-frequency antenna incorporated into the electronic board, wirelessly transmits the body temperature of the baby wearing the garment to a portable receiver unit equipped with a digital display.

Flores Canales [67] patented in 2015 a body temperature meter that comprises a temperature sensor, a computing device, and a connection interface between the temperature sensor and the computing device, where the interface transforms the sensor reading into an audio signal that can be processed by the computing device, all through the use of the invention method.

### Current Research and Developed Technologies for the Diabetic Foot in Spain

Current research in Spain related to diabetic foot is a focus on early detection of diabetic neuropathies, which can prevent the diabetic foot ulcers apparition [68]. Perception among 83% of general practitioners in Spain showed that management of diabetic foot ulcers is thought to be organized by multidisciplinary teams, with a presence of specialized podiatrists [69]. Actually, recent research shows that podiatrists work in multidisciplinary foot units, and implementing joint strategies with endocrinologists was associated to a 40% reduction in reulceration of diabetic foot [70]. Therefore, prevention of recurrent ulcers is feasible and should be a priority in a multidisciplinary diabetic foot unit.

Also in Spain, some teams are now trying to identify temperature thresholds, which allow for detecting further possible complications, such as plantar ulcers. In a sample of 277 diabetics with no foot complications, it was studied that any temperature change in the skin of the sole of the foot that is greater than $0.7{\;}^{\circ }$C (they considered four regions of interest, ROIs) calls for greater vigilance of the affected area [71]. This might be considered as the first value to implant vigilance of the affected area.

Other areas of interest are the relationship of how the foot posture could influence the temperature of the foot sole. In that way, although not directly related to diabetics, it seems that skin temperature is not related to foot eversion [72]. Thus, foot posture could not be an important determinant. However, there is scant literature in this field, and interesting lines of research are open. With a better classification of foot posture, i.e., using a foot posture index, it could be possible to identify ROIs exposed to more load, plantar pressures [73] and possibly, higher temperature. Therefore, monitoring these ROIs, we could detect better possible temperature changes that could influence the weakening of the skin sole and that could develop a plantar ulcer.

**Figure 4:**
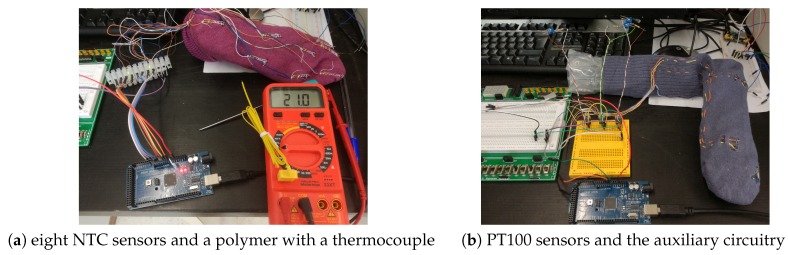
Prototype developed to collect temperature data: (**a**) sock developed with eight NTC sensors located on the sole of the foot, and a polymer with a thermocouple; (**b**) we set with PT100 sensors and the auxiliary circuitry to measure the PT100 with four wires.

However, most of these studies were based on temperatures taken with thermography procedures, which do not allow for obtaining continuous data. Obviously, in Spain, all types of sensors described in the previous section are well known [53,54,74,75]. However, this problem is challenging enough. Trying to develop a wearable to measure foot temperature, we tested some of those sensors [57]. One possibility was the creation of a sock with thermistors because they measure well, but the problem was that they were too bulky. Later, we continued with a montage with resistance thermometers; they are small, but they needed a lot of electronics to obtain the data correctly. Then, it is possible to propose the development of a new prototype with programmable electronic devices; they are very small and they measure well, the difficulty is that they are very small elements and it is necessary to solder wires for power and communications. Obviously, for the realization of our device, sensors of reduced size should be considered that do not cause discomfort in the person who uses them [57]. Figure 4 shows the sock developed there with eight NTC sensors located on the sole of the foot, in addition to the polymer that obtains temperatures with a thermocouple. In Figure 4b, we show the set with the PT100 sensors and the auxiliary circuitry to measure the PT100 sensors with four wires.

Once we were able to obtain the measures, it was necessary to change the microcontroller by an Arduino platform to get the data [76]. We decided to do it because of the simplicity in the assembly: with the microcontroller PIC, it is necessary to install all the auxiliary circuitry to work; however, with the Arduino platform, this is reduced to implement it on the same plate.

In addition, there is a large variety in Arduino plates and of different sizes. Once sensors are able to obtain the temperatures with a large plate, it is easy to repeat the process in a smaller plate, and it is possible to implement the whole system of data acquisition in a small box, easily portable by a person in the leg, at the same time that the communication with the mobile phone is made without any difficulty.

Arduino is an open platform based on a simple board with analog and digital inputs and outputs (I/O), and in a development environment that implements the Processing/Wiring language [76,77,78,79,80]. Arduino can be used in the development of autonomous interactive objects or it can be connected to a PC through the port using Flash, Processing, MaxMSP, etc. The open source IDE can be downloaded for free for Windows, Mac OS or Linux.

We have tested Arduino Mega 2560, a complex board since it is equipped with many inputs and outputs, both digital and analog as serial communication. It has 54 digital input/output pins (14 of which can be used as PWM outputs), 16 analog inputs, four UARTs (hardware serial ports), a 16 MHz crystal oscillator, USB connection, power jack, ICSP connector and reset button. It incorporates everything necessary for the microcontroller to work; we only need to connect it to a PC through a USB cable or with an external power supply. The Arduino Mega is compatible with most shields designed for Arduino Duemilanove, diecimila or UNO, which are other plates of the Arduino family. Arduino Mega uses an ATMega8U2 microcontroller. This allows higher transmission speeds through its USB port and does not require drivers for Linux or MAC (inf file is necessary for Windows). It also has the ability to be recognized by the PC as a keyboard, mouse, joystick, etc. The main features are: ATmega2560 microcontroller, input voltage from 7 to 12 V, 256 KB of flash memory and 16 MHz clock speed.

**Figure 5:**
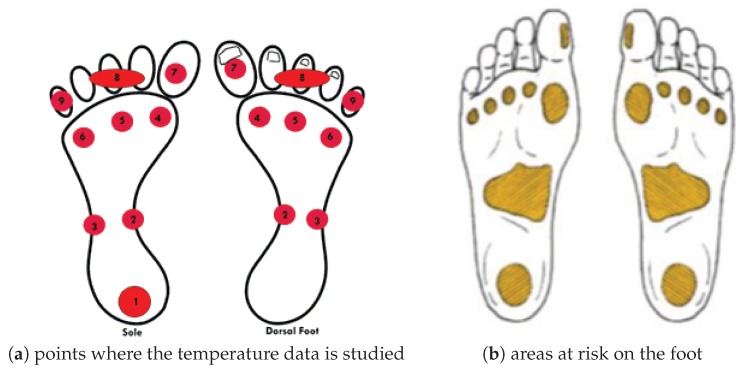
(**a**) we show a scheme with the points where the temperature data is studied: the plant and dorsal areas correspond to the same position, except for the number one that is only in the sole: (1) heel, (2) medial midfoot, (3) lateral mid-foot, (4) first metatarsal head, (5) central metatarsal heads, (6) fifth metatarsal head, (7) first finger, (8) central fingers, and (9) fifth finger; (**b**) there is an illustration with areas at risk on the foot taken from [82].

It is also convenient to analyze how many sensors the wearable will use and where we should place them. There is a previous study to obtain them [81]. These points are shown in Figure 5. Dendograms, heat maps and histograms given there provided some conclusions about where we should place sensors in case we cannot use nine sensors, but four instead, for example. These points are related with areas where it is very likely that a foot ulcer occurs according to some studies [82]. These areas are illustrated in Figure 5b. Detecting problems in these zones is of great interest. The smallest areas at risk are more or less a circle of 1 cm of diameter. This characteristic is of importance for systems built to detect problems in diabetic feet.

## 5. Conclusions

Diabetic patienes may suffer the diabetic foot problem that can be complicated by minor injuries and subsequent infection can lead to ischemic ulceration. In this study, we have carried out a review on wearables, medical devices, and different sensors specifically used for collecting vital data. We paid special attention to devices able to measure feet temperature. Nowadays, there are many Spanish wearables to measure vital signs such as pulse, blood pressure, blood oxygen, etc. However, diabetic wearables must be developed to measure temperature to improve the diabetic patient’s quality of life.

## Figures and Tables

**Table 1:** Wearable medical devices to measure vital data.

Device	Variables	Technology	Place
Motherboard™ [21]	Penetration of a proiectile, ECG, SpO${}_{2}$, temperature, voice	Intelligent garment	Used by the US Navy in combact
AMON (wrist–worn device) [34]	ECG, SpO${}_{2}$, Blood pressure, heart rhythm	Siemens TC35 Cellular Engine	Hospital or home
LifeGuard [35]	ECG, SpO${}_{2}$, activity, respiratory rate, heart rhythm, temperature	Bluetooth	remote and/or extreme environments
MagIC [36]	ECG, respiratory activity	Textile sensors	daily life and clinical environment.
Smart Vest [37]	ECG, PPG, body temperature, blood pressure, GSR, heart rate.	Home	wireless transceiver module (Xstream™)
LOBIN project [16]	ECG, temperature	RFID	Hospital

**Table 2:** Medical wearables specifically designed to get temperature data.

Device	Sensor Type	Technology	Place	Others
A grid for [41] feet temperature monitoring in bed	16 thermistors PBN-41E, Shibaura Denshi, Tokyo, Japan	data logger with a memory card	Bed	Measurement range: 0–$40{\;}^{\circ }$C Accuracy: $\pm 0.2^{\circ }$
Multipoint temperature [42] logger	LM35 as sensor and Arduino UNO	data logger with a memory card		Temperature Range: −55 to $150{\;}^{\circ }$C
A grid for feet temperature monitoring [43]	Temperature sensors	Multiple sensors to collect different vital data	Laboratory with the apparatus	

**Table 3:** Classification of thermocouples according to the voltage produced.

Type	Material	Voltage (mV)
B	Platinum–rhodium 30% vs. platinum–rhodium 6%	0 to 10.094
R	Platinum–rhodium 13% vs. platinum	0 to 16.035
S	Platinum–rhodium 10% vs. platinum	0 to 13.155
J	Iron vs. constantan	−7.89 to 39.130
K	Nickel–chromium vs. nickel	0 to 41.269
T	Copper vs. constantan	−5.60 to 14.86
E	Nickel–chromium vs. constantan	−9.83 to 53.11
